# Repeated static stretching was more effective for increasing range of motion and decreasing passive stiffness than a combination of a thermal agent with static stretching

**DOI:** 10.5114/biolsport.2026.161101

**Published:** 2026-05-20

**Authors:** Masatoshi Nakamura, Andreas Konrad, Kosuke Takeuchi, David G Behm

**Affiliations:** 1 Faculty of Rehabilitation Sciences, Nishi Kyushu University, 4490-9 Ozaki, Kanzaki, Saga, 842-8585, Japan; 2 Institute of Human Movement Science, Sport and Health, University of Graz, Graz, Austria; 3 Department of Physical Therapy, Kobe International University, Kobe-shi, Hyogo, Japan; 4 School of Human Kinetics and Recreation, Memorial University of Newfoundland, St. John’s, Newfoundland and Labrador, Canada

**Keywords:** Maximal voluntary isometric contraction, Microwave therapy, Stretch tolerance, Flexibility, Dorsiflexion, Heat

## Abstract

Combining static stretching (SS) and thermal agents could be beneficial for improving range of motion (ROM) and passive stiffness. However, in many previous studies, combining SS and thermal agents resulted in a longer intervention time than SS alone. Therefore, it is unclear whether the effects of SS alone and combining SS and thermal agents are similar when the total intervention time is standardized. This study aimed to compare the effects of SS alone and combine SS with thermal agents when the intervention time is standardized for 10 minutes. The participants were 14 healthy sedentary males (21.0 ± 0.4 y) in three conditions: 1) 120 s SS (480 s rest+120 s SS), 2) Heat+120 s SS (480 s thermal agent+120 s SS), and 3) 120 s SS × 4 times (120 s SS × 4 repetitions+40 s rest{ × 3 times}) conditions. The ankle dorsiflexion (DF) ROM, passive torque at DF ROM, passive stiffness, and plantar flexors maximal voluntary isometric contraction (MVIC) torque were measured before (PRE) and immediately after the intervention (POST). Two-way repeated-measures analysis of variance showed significant interaction effects in DF ROM and passive stiffness but not in passive torque at DF ROM and MVIC. Post-hoc tests showed that DF ROM in the 120 s SS × 4 times condition was significantly higher than in the 120 s SS condition, and the decrease in passive stiffness in the 120 s SS × 4 times condition was significantly higher than in the 120 s SS and the Heat+120 s SS conditions. In conclusion, repeating SS induced greater ROM increases and passive stiffness decreases than combining SS with thermal agents in a standardized intervention lasting 10 minutes.

## INTRODUCTION

In sports and rehabilitation settings, it is generally accepted that increasing muscle temperature before exercise is beneficial in preparation for sports or rehabilitation activities [1]. This can involve active or passive warm-ups utilizing thermal agents, such as hot packs, continuous therapeutic ultrasound, shortwave diathermy, and microwave applications. Previous research has demonstrated that a passive warm-up, such as the use of thermal agents, can increase range of motion (ROM) [1–4].

The increase in muscle temperature is thought to alter the viscoelastic properties of muscle and other collagenous tissue [5]. Previous studies have shown that thermal agents alone can decrease passive muscle stiffness [3, 6]. Also, previous studies showed that thermal agents combined with static stretching (SS) exercise could yield greater gains in ROM than SS alone [7, 8]. Fujita et al. investigated the combined effect of SS and thermal agents on the passive stiffness of the hamstrings muscle-tendon unit. However, they showed no superior effect of the combined SS and thermal agents on passive stiffness compared with SS alone [9]. Similarly, Nakamura et al. showed no superior effect of the combined effect on passive muscle stiffness of the quadriceps compared with SS alone [10]. The mechanism underlying the acute effects of ROM and stiffness gain from the combined use of thermal agents and SS remains controversial. In previous studies, mechanical properties, such as passive muscle stiffness, and perceptual sensations, such as pain and discomfort tolerance (i.e., stretch tolerance), have been implicated in increased ROM after SS [11, 12]. Previous studies suggested that increased ROM after thermal agents could also be attributed to changes in stretch tolerance [3, 4]. Therefore, changes in stretch tolerance could significantly affect ROM, even when combined with SS and thermal agents [9].

As mentioned above, a meta-analysis has shown that ROM gains are greater with combined SS and thermal agents than with SS alone [8]. Before this result can be applied in clinical and sports settings, time efficiency must be considered. The intervention time was not standardized in many previous studies investigating the combined effects of SS and thermal agents. Specifically, Fujita et al. compared the combined effect of 15 min of thermal agents and 3 min of SS with 3 min of SS alone. Therefore, if the intervention time for the combined effect of SS and thermal agents, and for SS alone, is standardized, it is unclear whether this significance will be maintained. When considering time efficiency, information on whether to use thermal agents in combination with SS or to repeat SS in situations where ROM and passive stiffness changes could be important for therapists and athletes. Thus, this study aimed to compare the effects of SS alone and SS combined with thermal agents, with the intervention time standardized to 10 minutes. We hypothesized that repeated SS could have superior effects on changes in ROM and passive stiffness when compared to combined SS with thermal agents, since there is a dose-response relationship between the SS duration and response to SS [13, 14].

## MATERIALS AND METHODS

### Experimental design

The study was conducted as a randomized, repeated-measures controlled experiment. Participants were instructed to visit the laboratory three times, with an interval of at least ≥ 48 h. Participants were exposed to three conditions in which the intervention time was standardized to 10 minutes: 1) 120 s SS (480 s rest +120 s SS), 2) Heat+120 s SS (480 s thermal agent+120 s SS), and 3) 120 s SS × 4 times (120 s SS × 4 repetitions+40 s rest between repetitions { × 3 times}) conditions using a computerized random number function in Microsoft Excel (Microsoft Corp., Washington, WA, USA) (Figure 1). All experimental conditions were performed at the same time of day (± 3 h) for each participant. The room temperature was controlled and maintained at approximately 24°C.

**FIG. 1:**
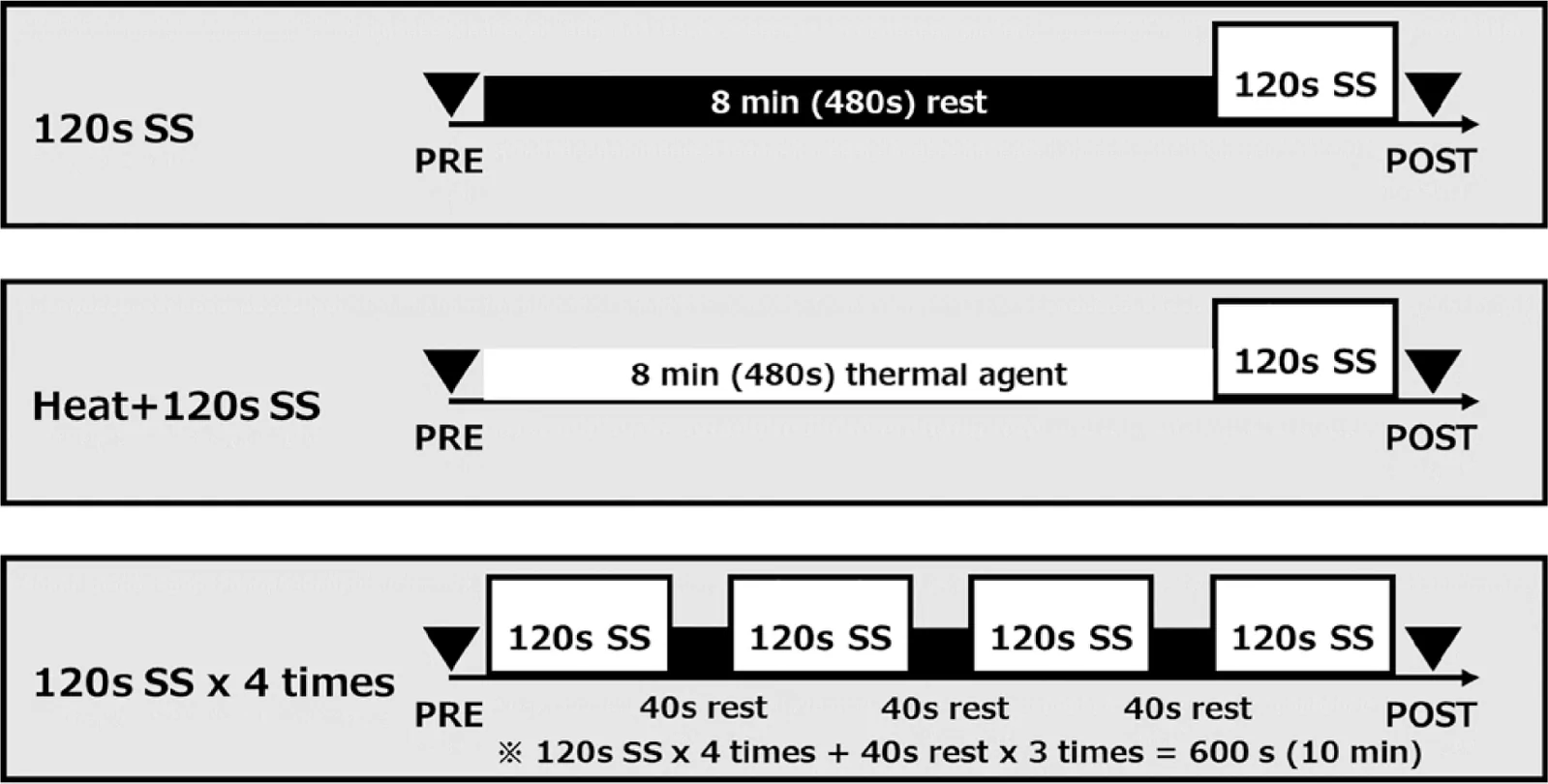
Experimental design Three conditions in which the intervention time was standardized to 10 minutes: 1) 120 s static stretching (SS) (480 s rest +120 s SS), 2) Heat+120 s SS (480 s thermal agent+120 s SS), and 3) 120 s SS × 4 times (120 s SS × 4 repetitions+40 s rest between repetitions {x 3 times}) conditions.

Outcome variables were measured in each condition before (PRE) and immediately after the intervention (POST). Dorsiflexors (DF) ROM, passive torque at DF ROM, passive stiffness, and plantar flexors (PF) MVIC torque were measured on the dominant side. In the 120 s SS conditions, after the PRE measurement, the participants rested in a seated position for 8 min (480 s), and then performed the 120 s SS (total duration: 600 s). In the Heat+120 s SS condition, participants receive 8 min (480 s) of hyperthermic stimulation using microwave therapy followed by 120 s of SS (total duration: 600 s). In the 120 s SS × 4 times condition, participants performed 120 s SS four times (480 s), with 40 seconds of rest between repetitions (total duration: 600 s).

### Participants

Fourteen healthy, sedentary males participated in this study (mean ± standard deviation (SD): age, 21.0 ± 0.4 years; height, 169.5 ± 3.9 cm; weight, 59.3 ± 10.2 kg). Participants with a history of neuromuscular disease or musculoskeletal injury involving the lower extremities were excluded. The required sample size for a repeated-measures two-way analysis of variance (ANOVA) (effect size = 0.40 [medium], α error = 0.05, and power = 0.80) using G*power 3.1 software (Heinrich Heine University, Düsseldorf, Germany) was at least 14 participants. The effect size in the sample was set based on the change in ROM according to a previous study [15].

Participants were thoroughly informed about the procedures and objectives of the study, after which they provided written informed consent. The study adhered to the requirements of the Declaration of Helsinki and was approved by the Ethics Committee of Nishikyushu University (Procedure: 24DRL25).

### Outcome assessment

#### DF ROM, passive torque at DF ROM, and passive stiffness

Each participant sat on a dynamometer chair (BIODEX system 3.0; BIODEX, Inc., Shirley, NY, USA), with the dominant leg fully extended at the knee joint and the ankle joint fixed to the footplate. The pelvis and distal thigh were fixed with a belt, and the hip flexion angle was set at 70° to prevent stretching of the sciatic nerve on the measurement side [16]. The footplate of the dynamometer was passively and isokinetically dorsiflexed at a speed of 5°/s from the neutral anatomical position to the DF angle just before the subject started to feel discomfort or pain [17–19]. Before the passive dorsiflexion assessment, two cycles of passive dorsiflexion were performed to familiarize the participant and to prevent a conditioning effect of passive stretching on muscle-tendon stiffness [20–22]. In addition, the investigator visually confirmed no heel displacement during passive stretching.

After participants completed the familiarization trials, they stopped the dynamometer by activating a handheld safety remote button when they started to feel discomfort or pain, and the angle just before this point was defined as the DF ROM. In addition, passive torque at DF ROM was defined as the stretch tolerance [11, 12]. Measurements were taken twice, and the average of these measurements was used for the analysis.

Based on a previous study [15, 23], we calculated passive stiffness from the passive torque at DF ROM at a certain angle. For the calculation, the passive torque was recorded over an angle range from 50% of the smallest DF ROM to the maximum DF ROM. The average of two measurements taken PRE and POST, respectively, was used in the analysis.

#### Maximum voluntary isometric contraction (MVIC) measurements

In a similar manner to the DF ROM measurements, each participant was seated on an isokinetic dynamometer chair at a 0° knee angle. PF MVIC was measured at the ankle joint in the neutral anatomical position (0°). After several (three or four repetitions) warm-up submaximal PF contractions, two MVICs were performed for 3-s each with 60-s intervals [15, 24]. We ensured that each participant could exert maximum muscle force within this 3-s period. Participants were strongly encouraged to make maximal efforts during contractions. The average value of the two MVICs was used for further analyses.

#### Static stretching (SS)

The SS intervention was performed using the dynamometer in the sitting position with the knee extended, as during the DF ROM and passive torque measurements. In this study, the footplate of the dynamometer was passively and isokinetically dorsiflexed at 5°/s from the neutral anatomical position to the angle just before participants felt discomfort or pain [17, 18]. Participants stopped the dynamometer by activating a safety trigger when they felt discomfort or pain, and the ankle joint was held at this angle for 120 s. The participant was instructed to be relaxed and keep their torso upright during stretching. The 120-s SS duration was adopted because a previous study reported it as the minimum SS duration required to decrease muscle stiffness [14].

#### Thermal agent intervention

The thermal agent intervention was performed with a microwave therapy device (Microwave Therapy ME-7250, OG Wellness Technologies Co., Ltd., Okayama, Japan) in a supine position, with the ankle joint relaxed on a treatment bed. The irradiation distance between the light and the plantar flexor muscles was 10 cm, and the power delivered was 80 W for 8 min. Participants were instructed to relax during the intervention.

### Statistical analysis

All statistical analyses were performed using SPSS (version 29.0, SPSS Japan Inc., Tokyo, Japan). All measurements were repeated twice, between 10 min sitting to assess test–retest reliability (10 healthy men; age, 21.1 ± 0.3 years; height, 176.1 ± 3.2 cm; body mass, 60.7 ± 10.5 kg), and test-retest reliability for each measure was assessed by calculating the intraclass correlation coefficients (ICCs).

The normal distribution of the data was confirmed using the Shapiro–Wilk test. A repeated-measures one-way ANOVA was conducted across three conditions to ensure consistency in PRE values. For all variables, a two-way repeated-measures ANOVA with two factors (test time [PRE vs. POST] and conditions [120 s SS vs. Heat+120 s SS vs. 120 s SS × 4]) was used to analyze both interaction and main effects. Effect sizes (ES) are presented as partial eta-squared values (ƞ_p_^2^) and categorized as either small effect (ƞ_p_^2^ < 0.01), medium effect (ƞ_p_^2^ = 0.02–0.14), or large effect (ƞ_p_^2^ > 0.14) [25]. Where applicable, post-hoc analyses were conducted using a paired t-test with Bonferroni correction to evaluate the differences between PRE and POST in each condition. ES (Cohen’s d) was calculated as the difference in the mean value divided by the pooled SD between PRE and POST in each condition. An ES of 0.00–0.19 was considered trivial, 0.20–0.49 as small, 0.50–0.79 as moderate, and ≥ 0.80 as large [25]. In addition, we calculated the percent change from PRE to POST for the outcome variables where a significant interaction effect was observed to compare the differences among the three conditions: percent change = (POST value − PRE value) / (PRE value) × 100. Because the Shapiro–Wilk tests showed that the percent change was not normally distributed, differences in the percent change among three conditions were determined using the Wilcoxon signedrank test with Bonferroni correction.

In addition, the relationship between the change in DF ROM and the change in passive torque at DF ROM and the passive stiffness in each condition was investigated. Since some data did not follow normality, we investigated the relationship using the Spearman’s rank correlation coefficient in each condition. A significance level of 5% was set, and all the results are shown as mean ± SD.

## RESULTS

The ICC values for DF ROM, passive torque at DF ROM, passive stiffness, and PF MVIC torque were 0.991 (95% confidence interval (CI): 0.965-0-0.997), 0.982 (95%CI: 0.939-0.994), 0.904 (95%CI: 0.716-0.968), and 0.993 (95%CI: 0.973-0.998), respectively.

Table 1 lists the changes in DF ROM, passive torque at DF ROM, passive stiffness, and PF MVIC torque in all conditions. There were no significant differences between the three conditions in any of the PRE variables. In addition, two-way repeated-measures ANOVAs showed significant (*P* < 0.05) interaction effects in DF ROM (*F* = 4.64, η_p_^2^ = 0.192) and passive stiffness (*F* = 16.2, η_p_^2^ = 0.453). The post-hoc test results showed that DF ROM experienced moderate to large magnitude significant increases (d = 0.45, 0.77, and 1.03 for 120 s SS, Heat+120 s SS s, 120 s SS × 4 times, respectively) and passive stiffness had small to large magnitude significant decreases (d = -0.28, -0.41, and -1.00, respectively) after intervention in all conditions. However, there were no significant interaction effects, but the main effects of time in passive torque at DF ROM and PF MVIC torque (*P* < 0.01, *F* = 62.8, η_p_^2^ = 0.617, *P* < 0.01, *F* = 31.5, η_p_^2^ = 0.447, respectively). Post hoc test showed that passive stiffness at DF ROM had small to moderate significant decreases (d = 0.39, 0.41, and 0.52, respectively) in all conditions and PF MVIC had small to moderate significant decreases in 120 s SS condition (d = -0.40) and 120 s SS × 4 times condition (d = -0.50), but not significant decreases in the Heat+120 s SS condition (*P* = 0.16, d = -0.21).

**TABLE 1:** The changes (mean ± SD) in dorsiflexion range of motion (DF ROM), passive torque at DF ROM, passive stiffness and plantar flexors (PF) maximal voluntary isometric contraction (MVIC) torque before (PRE), and after three intervention. The repeated-measures Two-way analysis of variance (ANOVA) results (TxC: interaction effect, T: time effect; *F*-value and partial η^2^ [η_p_^2^]) are shown in the right column. The Cohen’s d effect size (*d*) is also provided from the PRE values for all conditions.

condition	120 s SS		Heat + 120 s SS		120 s SS × 4 times		ANOVA results

	PRE	POST	PRE	POST	PRE	POST	P value, F value, η_p_^2^
DF ROM (deg)	19.7 ± 6.9 d =	22.7 ± 6.6* 0.45	18.3 ± 6.9 d =	24.0 ± 7.9* 0.77	17.3 ± 7.7 d =	24.6 ± 7.7* 1.03	T: p < 0.01, F = 86.2, η_p_^2^ = 0.688 C × T: p = 0.016, F = 4.64, η_p_^2^ = 0.192

Passive torque at DF ROM (Nm)	23.1 ± 8.2 d =	26.5 ± 9.6* 0.39	24.0 ± 7.9 d =	27.7 ± 10.4* 0.41	23.8 ± 9.2 d =	28.6 ± 9.4* 0.52	T: p < 0.01, F = 62.8, η_p_^2^ = 0.617 C × T: p = 0.474, F = 0.762, η_p_^2^ = 0.038

Passive stiffness (Nm/deg)	0.71 ± 0.18 d =	0.66 ± 0.14* -0.28	0.72 ± 0.18 d =	0.65 ± 0.17* -0.41	0.74 ± 0.19 d =	0.58 ± 0.14* -1.00	T: p < 0.01, F = 109.6, η_p_^2^ = 0.738 C × T: p < 0.01, F = 16.2, η_p_^2^ = 0.453
PF MVIC torque (Nm)	129.1 ± 23.4 d =	120.5 ± 19.3* -0.40	137.7 ± 23.6 d =	132.6 ± 25.1 -0.21	137.0 ± 32.8 d =	122.4 ± 25.2* -0.50	T: p < 0.01, F = 31.5, η_p_^2^ = 0.447 C × T: p = 0.08, F = 2.70, η_p_^2^ = 0.121

* : significant (*P* < 0.05) difference from the PRE value. SS: static stretching.

The comparison of the percentage change of DF ROM and passive stiffness are shown in Table 2. The Wilcoxon signed-rank test with Bonferroni correction showed that the percent change of DF ROM was significantly higher in the 120 s SS × 4 times condition than in the single 120 s SS condition (*P* = 0.024). However, there were no significant ROM differences between the 120 s SS condition and Heat+120 s SS condition (*P* = 0.123) and between the Heat+120 s SS condition and 120 s SS × 4 times condition (*P* = 0.474). Also, the Wilcoxon signed-rank test with Bonferroni correction showed that the percent change of passive stiffness in the 120 s SS × 4 times condition was significantly (*P* < 0.01) higher than the 120 s SS and the Heat+120 s SS conditions. However, no significant differences were observed between the 120 s SS and the Heat+120 s SS conditions (P = 0.252).

**TABLE 2:** The percent change of dorsiflexion range of motion (DF ROM) and passive stiffness (mean ± SD) among three conditions (120 s static stretching (SS) vs. Heat + 120 s SS vs. 120 s SS × 4 times conditions)

Condition	120 s SS	Heat + 120 s SS	120 s SS × 4 times
Change in DF ROM (%)	20.0 ± 19.0	36.1 ± 22.5	48.2 ± 37.7^*^
Change in passive stiffness (%)	−5.4 ± 6.5	−10.2 ± 6.5	−21.6 ± 5.2^*, #^

* : significant (*P* < 0.05) difference from the 120 s SS condition value

#* : significant (*P* < 0.05) difference from the Heat + 120 s SS condition value.

Significant positive correlations were found between DF ROM change and passive torque at DF ROM in the 120 s SS (*P* = 0.049, *r*_s_ = 0.534), Heat + 120 s SS (*P* < 0.01, *r*_s_ = 0.675), and 120 s SS × 4 times (*P* = 0.011, *r*_s_ = 0.653) conditions. But, no significant correlation was found between DF ROM change and passive stiffness change in the 120 s SS (*P* = 0.436, *r*_s_ = -0.226), Heat + 120 s SS (*P* = 0.375, *r*_s_ = 0.257), and 120 s SS × 4 times (*P* = 0.18, *r*_s_ = -0.38) conditions.

## DISCUSSION

We compared the acute effects of SS alone and combined SS with thermal agents when the intervention time was standardized for 10 minutes. The results showed that 8 min (640 s) of thermal agents before 120 s SS (total duration: 10 min) could increase DF ROM and stretch tolerance and decrease passive stiffness. Still, 4 repetitions of 120 s SS with 40 s rest between repetitions (total duration: 10 min) induced a relatively (%) and significantly larger passive stiffness change than a single bout of 120 s SS or combining 120 s of SS with a thermal agent. There have been many studies on the combined effects of SS and thermal agents; however, to the best of our knowledge, this is among the first to directly compare SS alone with thermal agents when the total intervention duration is standardized.

The current study showed that DF ROM and passive torque at DF ROM (stretch tolerance) significantly increased in all conditions. It was also revealed that the percent improvement in DF ROM was significantly greater in the 4 × 120 s SS condition than in the single 120 s SS condition. This is in accord with the reported SS dose-response relationship [13, 14]. However, there was no statistically significant difference (p = 0.474) when comparing 4 × 120 s SS to the combination of SS and a thermal agent (microwave therapy), which is consistent with the previous studies [9, 10]. Although there was no significant ROM difference between repeated SS and the combination of SS with microwave therapy, when considering simplicity, repeated SS could be as beneficial as adding the complication of purchasing and then setting up microwave therapy in combination with the SS.

The results suggested that the effect of changes in ROM and stretch tolerance could be similar whether or not a thermal agent is used in combination with a similar duration of SS. Therefore, if the goal is to increase ROM, it may be sufficient to perform a single bout of 120 s SS and with no need to add 8 min of microwave therapy (thermal agent).

Similarly, passive stiffness decreased significantly across all conditions, and the percent increase in passive stiffness was significantly greater in the 120 s SS × 4 times condition than in the 120 s SS and Heat+120 s SS conditions. These results support previous studies showing that combining SS with thermal agents has similar effects on reducing passive stiffness as SS alone [9, 10]. In addition, these results expand on the findings of previous studies and show that when the same amount of time is spent combining SS with thermal agents, the effect of decreasing passive stiffness is significantly greater when SS is repeated. Previous studies have reported that thermal agents can decrease passive stiffness [3, 6], while others have found no significant changes [9]. In detail, Nakamura et al. investigated the time-course change in passive muscle stiffness during hot pack application. They showed that the effect size was small (5 min: d = 0.29, 10 min: d = 0.31), although there was a statistically significant decrease in muscle stiffness. Therefore, adding an 8-min thermal agent to a 120-s SS to decrease passive stiffness did not provide additional benefits. As mentioned previously, based on the dose-response relationship between SS and passive stiffness [13, 14], repeating the 120 s of SS was more effective for decreasing passive stiffness than the other conditions.

Regarding PF MVIC torque decreases, there was a main effect of time but no interaction effect. It is well known that prolonged SS can cause a decrease in maximum muscle strength [26–28]. A recent review by Warneke and Lohmann [29] was in agreement with the prior reviews, indicating that more than 60 s SS could cause a significant decrease in muscle strength. In agreement with these prior reviews, SS of 120 s or 480 s (4 × 120 s) in the present study induced significant small to moderate magnitude impairments in PF MVIC torque.

This study investigated the acute effects of combining SS and a thermal agent on ROM, passive stiffness, and MVIC torque from the perspective of standardizing the intervention duration. As a result, when considering the aspect of time efficiency, the 120 s SS significantly increased ROM and passive stiffness. Moreover, when the aim is to increase the effect of this 120 s, it is more effective to repeat the SS rather than use a thermal agent. In particular, regarding passive stiffness, theoretically, when the stiffness of the muscletendon unit is high, the demands for energy absorption and release might rapidly surpass the capacity of the muscle-tendon unit, potentially increasing the risk of injuries [30, 31]. Furthermore, passive muscle stiffness could also be related to muscle injury [32]. Therefore, the results of this study could be useful information for athletes and therapists aiming to prevent or reduce the recurrence of muscle injury. However, since we only investigated the acute effect, it is unclear whether longer-duration SS leads to the prevention of muscle injury and a reduction in recurrence over the long-term intervention, so this is a topic for future study.

There were some limitations in this study. Because of the nature of interventions, it was not feasible to blind participants or intervention providers, which may introduce bias. Since this study targeted healthy young men, it is necessary to conduct further studies targeting other populations, such as females, athletes, injured athletes, and other age groups (i.e., youth and seniors). In addition, the mechanisms of changes in ROM, passive stiffness, and MVIC torque in this study are unclear. The previous study indicated that the increase in DF ROM could be involved with a change in passive torque at DF ROM, i.e., changes in stretch tolerance after SS and thermal agent [9]. In this study, no correlation was found between changes in DF ROM and changes in passive stiffness, but a significant positive correlation was found between changes in DF ROM and passive torque at DF ROM (r_s_ = 0.534, 0.675, and 0.653 in each condition). Therefore, it was possible that changes in stretch tolerance might be related to changes in ROM, but further investigation is needed to determine the details. In addition, this study only measured the effects immediately after the intervention, and the prolonged (sustained) effects remain unknown. Moreover, this study used a 10-minute time period. Considering its application in sports and rehabilitation settings, it is necessary to conduct further studies with prolonged (sustained) effect and different time allocations and shorter periods to establish an effective SS protocol that can effectively change ROM and passive stiffness.

## CONCLUSIONS

In conclusion, our results revealed that an 8 min application of microwave therapy (thermal agent) before 120 s SS could induce increases in DF ROM, stretch tolerance, and decreases in passive stiffness. Still, repeating SS in the same intervention duration caused a particularly larger passive stiffness decrease than combined SS with a thermal agent. Thus, repetition of SS is recommended to efficiently induce changes in passive properties in rehabilitation and sports settings.

## Data Availability

All data generated or analyzed during this study are included in the article.
